# Curcumin Induces Cell Death and Restores Tamoxifen Sensitivity in the Antiestrogen-Resistant Breast Cancer Cell Lines MCF-7/LCC2 and MCF-7/LCC9

**DOI:** 10.3390/molecules18010701

**Published:** 2013-01-08

**Authors:** Min Jiang, Ou Huang, Xi Zhang, Zuoquan Xie, Aijun Shen, Hongchun Liu, Meiyu Geng, Kunwei Shen

**Affiliations:** 1 Comprehensive Breast Health Center, Ruijin Hospital, Shanghai Jiao Tong University School of Medicine, Shanghai 200025, China; E-Mails: jiangmin1023@126.com (M.J.); ou_huang@163.com (O.H.); zhangxi0714@yeah.net (X.Z.); 2 Division of Antitumor Pharmacology, Shanghai Institute of Materia Medica, Chinese Academy of Sciences, Shanghai 201203, China; E-Mails: zuoquan_xie@163.com (Z.X.); aijun_shen@163.com (A.S.); hongchun_liu@126.com (H.L.)

**Keywords:** curcumin, endocrine resistance, tamoxifen, NF-κB, Src/FAK, Akt/mTOR, EZH2, cyclin D1, c-Myc

## Abstract

Curcumin, a principal component of turmeric (*Curcuma longa*), has potential therapeutic activities against breast cancer through multiple signaling pathways. Increasing evidence indicates that curcumin reverses chemo-resistance and sensitizes cancer cells to chemotherapy and targeted therapy in breast cancer. To date, few studies have explored its potential antiproliferation effects and resistance reversal in antiestrogen-resistant breast cancer. In this study, we therefore investigated the efficacy of curcumin alone and in combination with tamoxifen in the established antiestrogen-resistant breast cancer cell lines MCF-7/LCC2 and MCF-7/LCC9. We discovered that curcumin treatment displayed anti-proliferative and pro-apoptotic activities and induced cell cycle arrest at G2/M phase. Of note, the combination of curcumin and tamoxifen resulted in a synergistic survival inhibition in MCF-7/LCC2 and MCF-7/LCC9 cells. Moreover, we found that curcumin targeted multiple signals involved in growth maintenance and resistance acquisition in endocrine resistant cells. In our cell models, curcumin could suppress expression of pro-growth and anti-apoptosis molecules, induce inactivation of NF-κB, Src and Akt/mTOR pathways and downregulate the key epigenetic modifier EZH2. The above findings suggested that curcumin alone and combinations of curcumin with endocrine therapy may be of therapeutic benefit for endocrine-resistant breast cancer.

## 1. Introduction

Breast cancer is the most commonly diagnosed malignancy and the second leading cause of cancer mortality among women in the United States [1]. About 70% breast cancers are classified as estrogen receptor (ER) positive and could be treated with antiestrogens [2,3]. Tamoxifen, a selective ER modulator (SERM), as the gold standard of endocrine therapy, has been introduced for neoadjuvant and adjuvant treatment of ER positive breast cancer patients [4,5]. Unfortunately, nearly 40% of breast cancer patients develop *de novo* or acquired resistance after 1–3 years of tamoxifen therapy [6,7]. Therefore, improved treatment strategies for ER positive antiestrogen-resistant breast cancer are urgently needed. 

Studies have already revealed several mechanisms of tamoxifen resistance, including: (i) alteration of ER-associated transcription factors and co-activators, such as NF-κB [8] and AP1 [9]; (ii) activation of RTK signaling, such as Src [10] and PI3K/Akt [11]; (iii) altered regulation of downstream signaling, such as cyclin D1, c-Myc [12], p21 [13] and Bcl-2 [14]; (iv) activation of RTKs, such as ERBB2 [15] and IGF1R [16]. These studies have led to various treatment strategies combining tamoxifen and targeted inhibitors. However, not all clinical trials of targeted inhibitors in combination with tamoxifen have yielded consistent favourable results [17]. It is estimated that inhibition by a single molecule cannot suppress the cross-talk and negative feedback loops in the complex cellular networks. Therefore, multi-target drugs have been accepted as the current trend in drug design and discovery for killing endocrine-resistant breast cancer cells and overcoming the endocrine resistance.

Curcumin, an effective anticancer component from turmeric, regulates multiple targets and has been used to enhance targeted therapy sensitization and to assist chemotherapy in treating breast cancer [18,19]. Curcumin has also been used as a therapeutic agent for reversing resistance to chemotherapy by inhibition of NF-κB signaling pathway [20]. There is compelling evidence that curcumin modulates multiple pathways in breast cancer, including PI3K/Akt, MAPK, NF-κB, cyclin D, c-Myc, *etc*. [21]. These molecular targets of curcumin are also known to be involved in growth maintenance and resistance acquisition in antiestrogen-resistant breast cancer.

In this study, we treated endocrine-resistant MCF-7/LCC2 and MCF-7/LCC9 breast cancer cells with curcumin to induce growth inhibition and to resensitize cells to tamoxifen. Furthermore, to explore the potential clinical application of curcumin, we analyzed the underlying mechanisms of curcumin in cytotoxicity and the reversal of endocrine resistance in breast cancer.

## 2. Results and Discussion

### 2.1. Results

#### 2.1.1. Curcumin Suppresses Proliferation of MCF-7/LCC2 and MCF-7/LCC9

**Figure 1:**
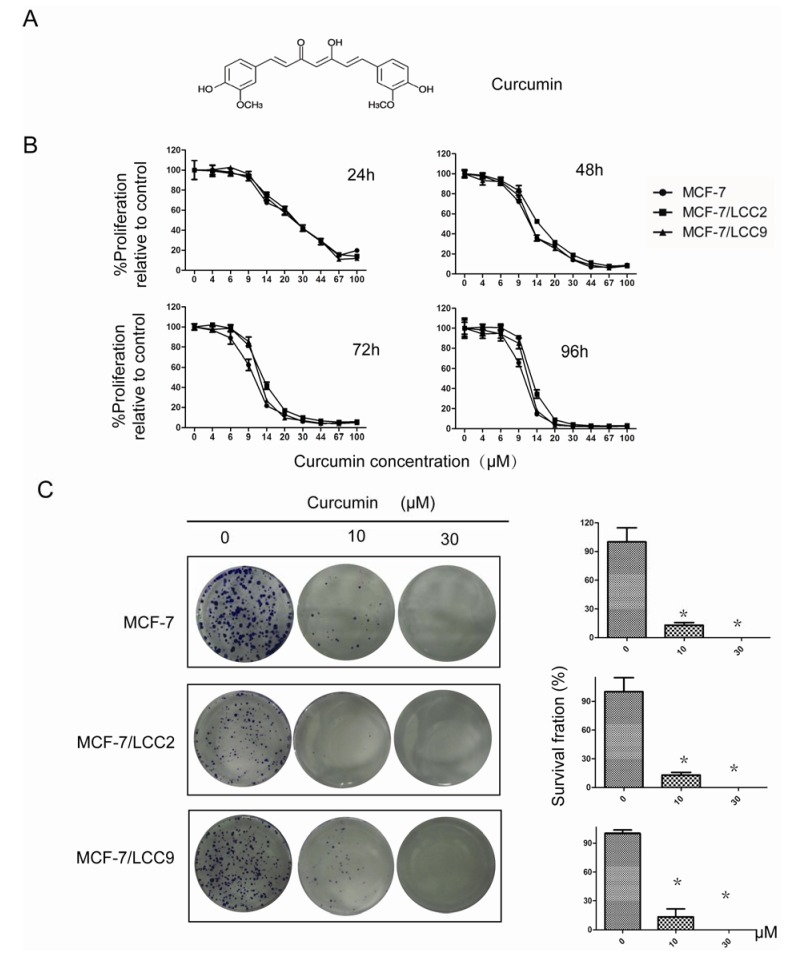
Curcumin inhibits proliferation of MCF-7/LCC2 and MCF-7/LCC9. (**A**) The chemical structure of curcumin; (**B**) Cells were treated with varying concentrations of curcumin, cell proliferation and IC50 were determined by SRB assay on days 1, 2, 3 and 4. Each value represents the mean ± SD (n = 3); (**C**) Curcumin suppressed colony formation in endocrine resistant cells. Cell were treated with curcumin (10 and 30 μM) and were allowed to form colonies in fresh medium for 14 days. Photomicrographic difference (Left panel) and influence of cell number (mean ± SD. n = 3) (Right panel) in colony forming were shown. Asterisks indicate *p* < 0.05.

To examine the biological effect of curcumin, MCF-7/LCC2, MCF-7/LCC9 and their wild-type cells were treated with different concentrations of curcumin for 96 h. Cell proliferation changes were evaluated with a SRB assay. As shown in Figure 1B, in MCF-7/LCC2 and MCF-7/LCC9 cells, cell proliferation was inhibited by curcumin in a dose-dependent manner. This anti-proliferation effect was observed within a 24 h period, which continued to increase over the next 96 h. Similar IC_50_ values for curcumin in endocrine-resistant cells and the wild-type cells were detected (IC_50_ = 9.718 μM after 96 h or 9.815 μM after 48 h, 12.240 μM after 96 h or 12.004 μM after 48 h and 11.344 μM after 96 h or 10.930 μM after 48 h in MCF-7, MCF-7/LCC2 and MCF-7/LCC9 cells, respectively). These results indicate that curcumin has potent antiproliferation effect in breast cancer cells, whether or not they have resistance to antiestrogen therapy. We also found HBL-100 was insensitive to curcumin inhibition compared with breast cancer cells (Supplementary Information Figure S1A).

#### 2.1.2. Curcumin Inhibits the Colony-Forming Ability of MCF-7/LCC2 and MCF-7/LCC9

To determine the long-term effect of curcumin on cell growth, cells were treated with 10 μM and 30 μM curcumin for 24 h. After that, the cells were cultured in fresh media without curcumin for two weeks. As shown in Figure 1C, curcumin treatment remarkably reduced colony formation capability. At 10 μM of curcumin, the number of colonies was significantly decreased (*P *< 0.05) from 162 to 22 in MCF-7/LCC9 cells, from 85 to 7 in MCF-7/LCC2 cells and from 201 to 26 in wild-type cells. Colony formation was almost completely suppressed by 30 μM curcumin.

#### 2.1.3. Curcumin Induces Apoptosis in MCF-7/LCC2 and MCF-7/LCC9

**Figure 2:**
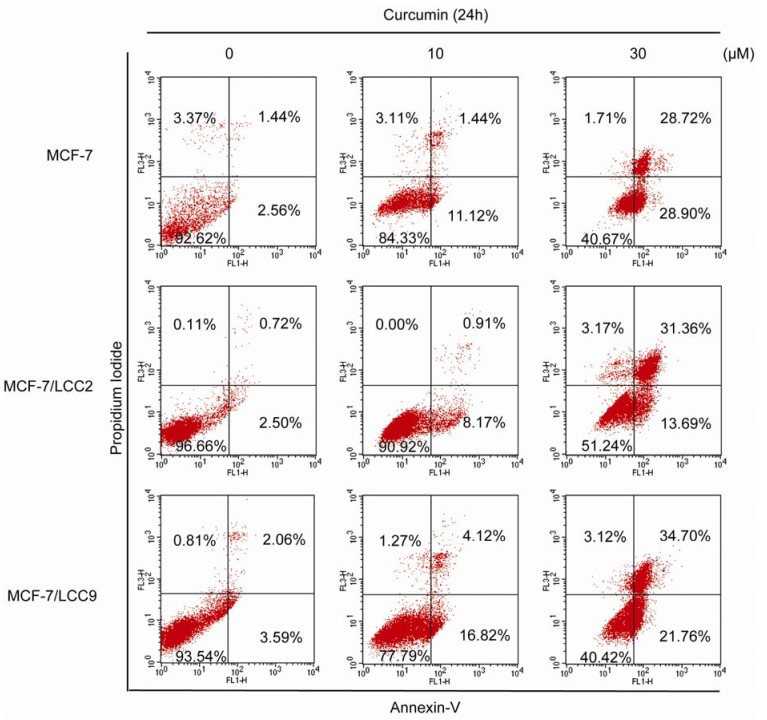
Curcumin induces apoptosis in MCF-7/LCC2 and MCF-7/LCC9. Cells were incubated with curcumin (10 and 30 μM) for 24 h, followed by staining with Annexin-V/PI. The lower right quadrant (Annexin-V+/PI−) and upper right quadrant (Annexin-V+/PI+) indicate the percentage of early apoptosis and late apoptosis.

We used Annexin-V/PI staining and flow cytometry to examine whether curcumin-induced growth inhibition was a result of apoptosis. Our results showed that curcumin in a dose-dependent manner induced apoptosis in endocrine resistant and wild-type cells (Figure 2), but not in HBL-100 (Supplementary Information Figure S1B). In cells exposed to 10 μM curcumin, few of them were PI positive, indicating that low doses of curcumin did not induce necrosis. However, treatment with 10 μM curcumin resulted in an increase of cells undergoing early apoptosis. The percentage of Annexin-V+/PI− cells was increased from 2.56% to 11.12%, from 2.50% to 8.17% and from 3.59% to 16.82% in MCF-7, MCF-7/LCC2 and MCF-7/LCC9 cells. Under the treatment with a higher dose at 30 μM, the Annexin-V+/PI− cells increased to 28.90%, 13.69% and 21.76% in MCF-7, MCF-7/LCC2 and MCF-7/LCC9 cells. Moreover, 30 μM curcumin caused a significant increase, 28.72% in MCF-7, 31.36% in MCF-7/LCC2 and 34.70% in MCF-7/LCC9, in the percentage of late apoptotic cells. Thus, high doses of curcumin induced both apoptosis and necrosis in endocrine resistant cells.

#### 2.1.4. Curcumin Induces G2/M Phase Arrest in MCF-7/LCC2 and MCF-7/LCC9

**Figure 3:**
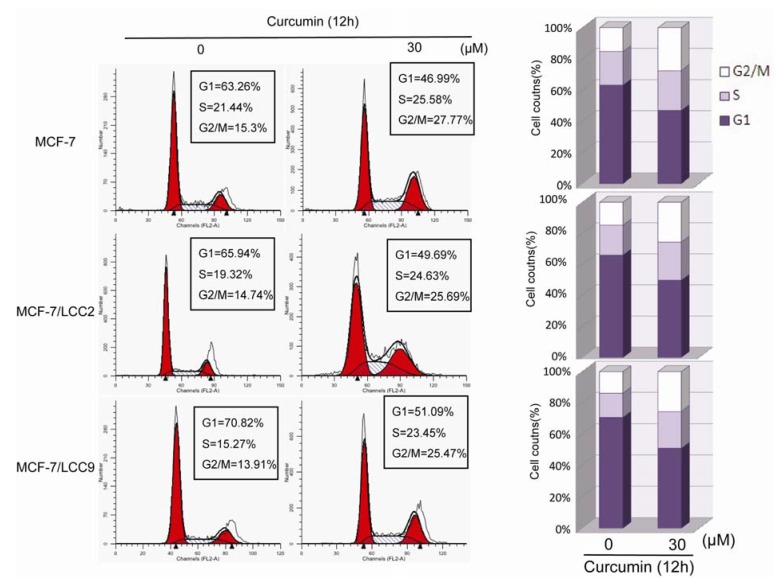
Curcumin induces G2/M phase arrest in MCF-7/LCC2 and MCF-7/LCC9. Cells were treated with 30 μM for 12 h and DNA content was analyzed by flow cytometry. The percentage of cells in G1, S and G2/M of cell cycle are shown. These results were from one representative experiment of three independent trials.

To further evaluate the effect of curcumin on cell growth, we tested the effect of 30 μM curcumin on the cell cycle distribution of endocrine resistant cells. As shown in Figure 3, in comparison with DMSO-treated cells, curcumin induced an accumulation of cells in the G2/M phase fraction with concomitant reduction of cell numbers in G1 phase. The G2/M phase fraction increased from 15.3% to 27.77% in wild-type cells, from 14.74% to 25.69% in MCF-7/LCC2 cells and from 13.91% to 25.47% in MCF-7/LCC9 cells. Consistent with the effect of curcumin on HBL-100 proliferation and apoptosis, curcumin induced only 3% increase in G2/M phase fraction (Supplementary Information Figure S1C). Thus, curcumin induces G2/M phase arrest in antiestrogen sensitive and resistant breast cancer cells.

#### 2.1.5. Curcumin Sensitizes Tamoxifen Action in MCF-7 and Reverses Tamoxifen Resistance in MCF-7/LCC2 and MCF-7/LCC9

To determine whether curcumin are able to show a synergistic effect on the grow inhibition with tamoxifen in MCF-7 cells, cells were exposed to curcumin and tamoxifen (alone or in combination) for three days, followed by treatment of tamoxifen alone for another four days. Dose response survival curves recorded over a range of OHT (4-hydroxytamoxifen) concentration from 0.5 to 8 μM showed that curcumin led to an increase in the sensitivity of MCF-7 cells to OHT compared with OHT alone treatment. The IC_50_ in curcumin treated cells was <0.5 μM compared with 1.22 μM for control-treated cells, resulting in a more than 2.4-fold increase in sensitivity to OHT. 

**Figure 4:**
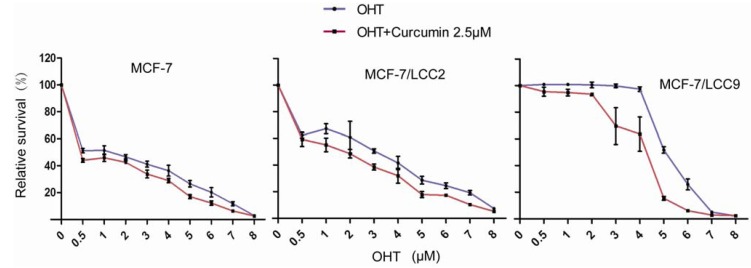
Curcumin enhances the sensitivity of MCF-7 and endocrine resistant cells (MCF-7/LCC2 and MCF-7/LCC9) to OHT. Cells were treated with combination of increasing concentrations of OHT and 2.5 μM curcumin for 3 days and OHT alone for the next 4 days. Drug response was determined by SRB assay. Each value represents the mean ± SD (n = 3).

To examine the biological effect of curcumin in endocrine resistance, tamoxifen-resistant MCF-7 cells (MCF-7/LCC2 and MCF-7/LCC9 cells) were examined. As shown in Figure 4, survival curves recorded over a range of OHT concentrations from 0.5 to 8 μM confirmed that the cells indeed were resistant to OHT. After receiving the same curcumin regimen as that for MCF-7 cells, the IC_50_ of OHT was decreased from 3.30 μM to 1.78 μM in MCF-7/LCC2 and from 5.15 μM to 4.09 μM in MCF-7/LCC9 cells. Thus, curcumin induced an increase in sensitivity to OHT in endocrine cells.

When the normal breast epithelial cell line (HBL-100) was treated with combinations of OHT and curcumin, no significant growth inhibition was found (Supplementary Information Figure S1D).

#### 2.1.6. Curcumin Suppresses the Protein and mRNA Expression of Cell Signals Associated with Proliferation in MCF-7/LCC2 and MCF-7/LCC9

**Figure 5:**
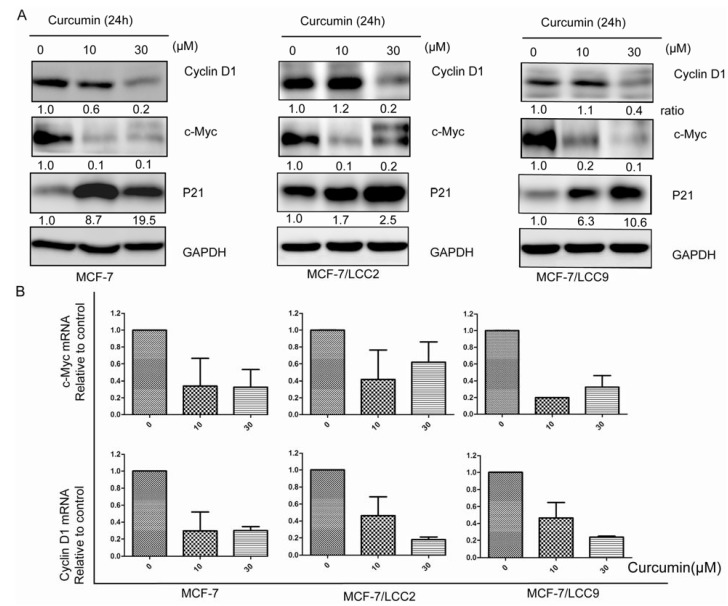
Curcumin downregulates pro-proliferation molecules in protein and mRNA levels in MCF-7/LCC2 and MCF-7/LCC9. (**A**) All cells treated with curcumin (10 and 30 μM) for 24h and then subjected to western blotting using anti-c-Myc, anti-cyclin D1 and anti-p21 antibodies; (**B**) Real-time PCR analysis of total RNA from wild-type and resistant cells following 10 and 30 μM of curcumin treatment induced decrease in expression of c-Myc and cyclin D1 mRNA. Data are presented as mean ± SD (n = 3).

To examine whether suppression of proliferation of endocrine resistant cells by curcumin is due to downregulation of molecules involved in cell proliferation, we tested the protein and mRNA levels of cyclin D1 and c-Myc in cells. We found that curcumin treatment inhibited the expression of mRNA and protein in cyclin D1 and c-Myc in a dose-dependent manner (Figure 5). Compared with tamoxifen-sensitive cells, MCF-7/LCC9 cells expressed increasing level of c-Myc while MCF-7/LCC2 cells expressed higher level of cyclin D1. Furthermore, 10 μM curcumin treatment had a minor effect on cyclin D1 protein level, but caused a dramatic decrease in mRNA levels. However, curcumin at a higher concentration of 30 μM diminished cyclin D1 expression in both protein and mRNA levels. Moreover, 10 μM curcumin was sufficient to inhibit c-Myc expression in protein and mRNA levels. After the same curcumin regimen treatment, expression of p21 protein was increased in wild-type and endocrine resistant cells. 

#### 2.1.7. Curcumin Downregulates the Protein and mRNA Expression of Cell Survival Signals in MCF-7/LCC2 and MCF-7/LCC9

**Figure 6:**
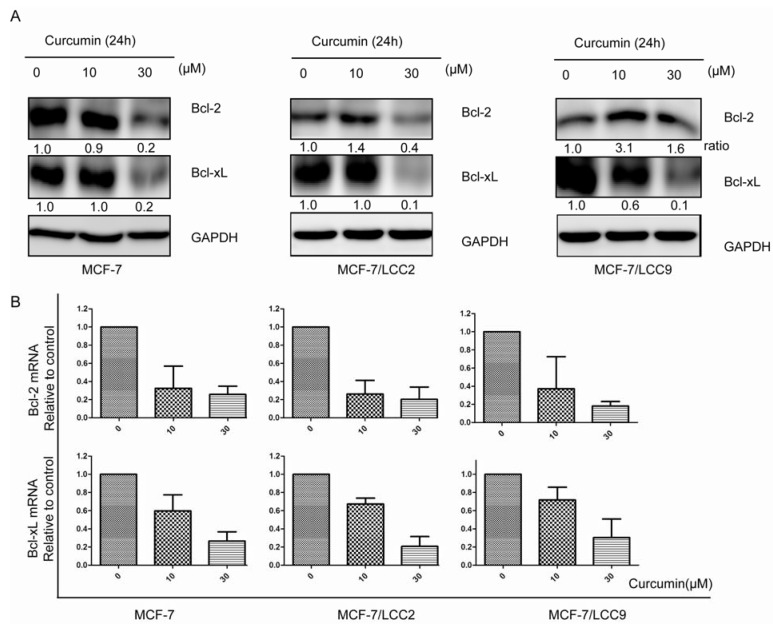
Curcumin suppresses cell survival signals in protein and mRNA levels in MCF-7/LCC2 and MCF-7/LCC9. (**A**) Lysates from curcumin (10 and 30 μM) treatment for 24h with western blot revealed significant reduction in expression of Bcl-2 and Bcl-xL protein levels in sensitive and resistant cells; (**B**) Real-time PCR showed the similar trend in downregulation of Bcl-2 and Bcl-xL in mRNA levels. Data are presented as mean ± SD (n = 3).

We further investigated the mechanisms underlying curcumin-induced apoptosis in endocrine resistant cells. As shown in Figure 6, Bcl-2 and Bcl-xL mRNA levels were significantly down-regulated by curcumin in both tamoxifen-sensitive cell line (MCF-7) and tamoxifen-insensitive cell lines (MCF-7/LCC2 and MCF-7/LCC9) in a dose-dependent manner. Western blotting also demonstrated that curcumin reduced Bcl-2 and Bcl-xL protein levels significantly at the concentration of 30 μM.

#### 2.1.8. Curcumin Inhibits IKK-NF-κB Signaling Pathway in MCF-7/LCC2 and MCF-7/LCC9

**Figure 7:**
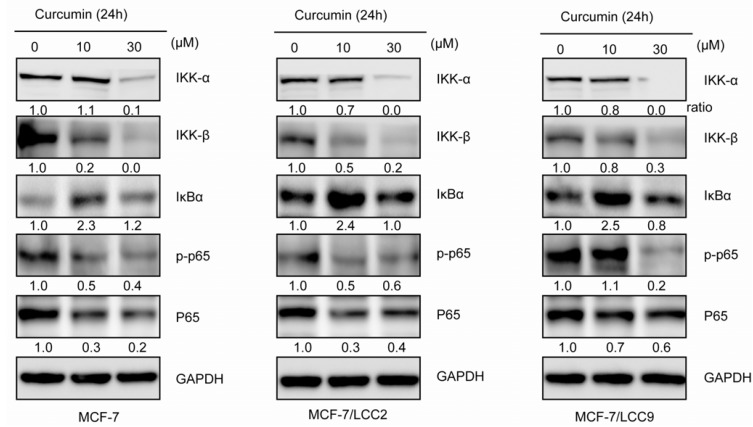
Curcumin inhibits IKK-NF-κB signaling pathway in MCF-7/LCC2 and MCF-7/LCC9. Cells were incubated with curcumin (10 and 30 μM) for 24h and then analyzed by western blot for IKKα, IKKβ, IκBα, p65 and p-p65 (Ser536).

Since IKK-NF-κB has been reported to be globally involved in regulation of cell survival, proliferation and endocrine resistance, we investigated the state of IKK-NF-κB signaling pathway. As shown in Figure 7, IKKα and IKKβ was expressed at similar levels in different cell lines. The key protein NF-κB p65 was likewise expressed at similar levels in three cell lines. We further examined the phosphorylation status of p65 in three cell lines. High levels of p65 phosphorylation were detected in MCF-7/LCC9, while MCF-7/LCC2 expressed only slightly higher levels of phospho-Ser536-p65 compared with MCF-7 cells. 

When treated with curcumin, p65 protein decreased significantly, followed by a decline in p65 phosphorylation in MCF-7 and MCF-7/LCC2 cells. However, in MCF-7/LCC9 cells, curcumin slightly suppressed p65 expression but abolished its phosphorylation. The maximum suppression effect was observed at a concentration of 30 μM. NF-κB phosphorylation is known to require the degradation of IκBα and activation of IKKs. In our study, curcumin significantly suppressed the degradation of IκBα in MCF-7/LCC2 and MCF-7/LCC9 cells. The degradation of IκBα is mediated through activation of IKKs. Therefore, we examined the expression of IKKα and IKKβ in endocrine resistant and sensitive cells. We found that curcumin suppressed the protein levels of both IKKα and IKKβ.

#### 2.1.9. Curcumin Inhibits Src Activation and Downregulates FAK Expression in MCF-7/LCC2 and MCF-7/LCC9

**Figure 8:**
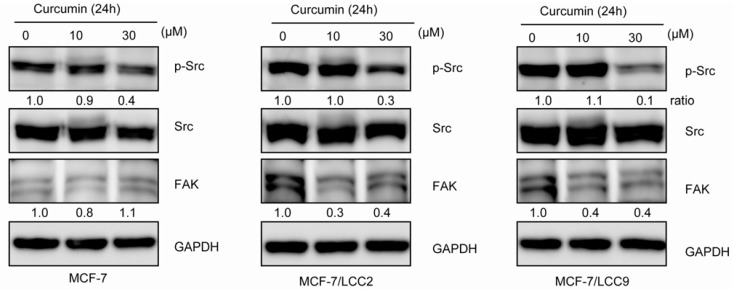
Curcumin inhibits Src/FAK in MCF-7/LCC2 and MCF-7/LCC9. Cells were treated with curcumin (10 and 30 μM) for 24 h and the protein expression was detected by anti-FAK, anti-Src and anti-p-Src(Tyr418).

Since Src/FAK activation has been reported to be involved in acquisition of resistance of breast cancer to antiestrogen therapy, we examined the Src/FAK state in cells. As shown in Figure 8, we were essentially unable to detect expression of FAK in endocrine sensitive cells, whereas FAK was highly expressed in MCF-7/LCC2 and MCF-7/LCC9 cells. When receiving curcumin treatment, the FAK expression was significantly suppressed. Although no change of total Src expression was detected between the cell lines, our study revealed the phosphorylation of Src(Tyr418) was increased in resistant cells compared with their sensitive counterparts. However, curcumin treatment significantly decreased phosphorylation of Src in endocrine resistant cells. 

#### 2.1.10. Curcumin Inhibits Akt/mTOR Signaling in MCF-7/LCC2 and MCF-7/LCC9

**Figure 9:**
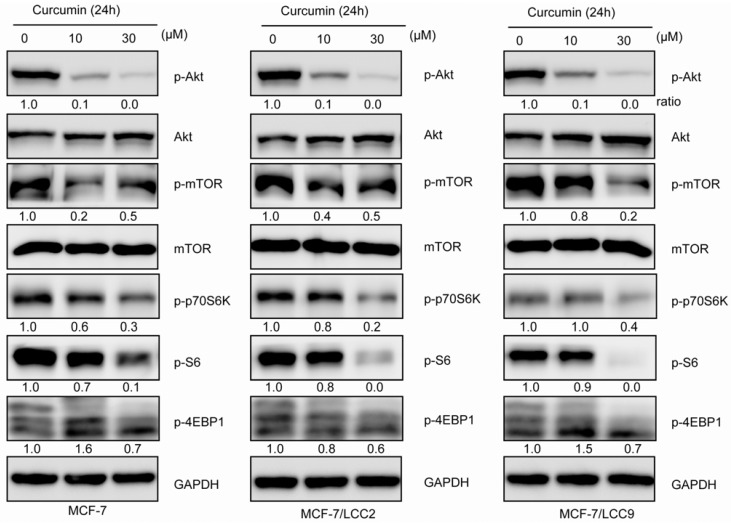
Curcumin inhibits Akt/mTOR pathway in MCF-7/LCC2 and MCF-7/LCC9. Cells were incubated with indicated concentrations of curcumin and then analyzed by western blot for detecting Akt, p-Akt (Thr308), mTOR, p-mTOR(Ser2488) and their downstream signals: 4EBP1, p-4EBP1(Thr37/46), S6, p-S6 (Ser235/236), p70S6K and p-p70S6K (Thr389). Data about 4EBP1, S6 and p70S6K was not shown.

We also examined the effects of curcumin on the Akt/mTOR signaling in resistant and sensitive cell lines. As shown in Figure 9, curcumin inhibited the phosphorylation of Akt(Thr308), mTOR(Ser2488), p70S6K(Thr389), S6(Ser235/236) and 4EBP1(Thr37/46) in a similar dose-dependent manner. Curcumin, at the concentration of 30 μM could abolish the activation of Akt/mTOR pathway. In all experiments, the total Akt, mTOR, p70S6K, S6 and 4EBP1 were showed no significant change.

#### 2.1.11. Curcumin Suppresses the Expression of EZH2 and Induces ERK Activation in MCF-7/LCC2

**Figure 10:**
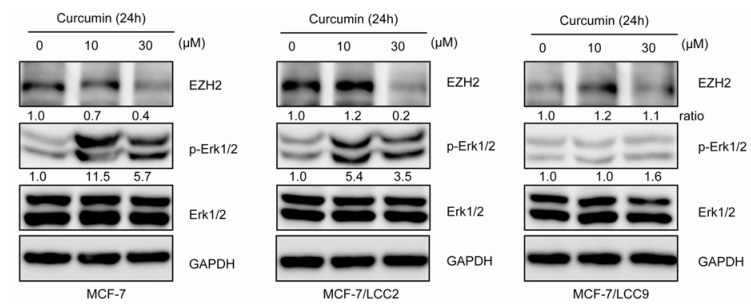
Curcumin inhibits EZH2 expression in MCF-7/LCC2. After treatment of indicated concentrations of curcumin for 24 h, cells were harvested and analyzed by western blot using antibodies to Erk1/2, p-Erk1/2 (Thr202/Tyr204) and EZH2.

To determine whether the effects of curcumin involved the regulation of EZH2, cells were treated with curcumin at 10 and 30 μM for 24h. The results, shown in Figure 10, demonstrated that curcumin decreased the expression of EZH2 in a concentration-dependent pattern in MCF-7 and MCF-7/LCC2. However, MCF-7/LCC9 cells expressed low levels of EZH2, which was almost not subject to the curcumin. Since previous studies had demonstrated that curcumin exerted biological effects by activating the ERK in breast cancer cells, we also investigated the ERK state in the present study. Curcumin treatment could significantly increased the ERK1/2 phosphorylation in MCF-7 and MCF-7/LCC2 but not in MCF-7/LCC9 cells.

### 2.2. Discussion

In the present study, we have demonstrated the effects of curcumin, a polyphenol derived from the rhizome of the perennial herb *Curcuma longa*, on cell proliferation, apoptosis, cell cycle and tamoxifen sensitivity in antiestrogen resistant cells. In the literature, multiple mechanisms of action of curcumin related to PI3K/Akt, MAPK, NF-κB,β-catenin and ROS in breast cancer have been shown [21]. It is important to clarify the mechanisms of action because it may lead to new drug for intervention in therapy of endocrine resistant breast cancer. We hypothesized that curcumin could affect the biological actions of antiestrogen-resistant breast cancer cells. In the present study, in addition to cell death induction, we discovered that curcumin could concurrently reverse resistance in endocrine-resistant cells and increase sensitivity in sensitive cells. Indeed, the concurrent abilities of resistance reversal and sensitization may be beneficial to ER positive breast cancer comprising heterogeneous cells. We furthermore elucidated that curcumin reversed the resistance by inhibiting the signaling pathways involved in acquisition of antiestrogen resistance.

Tamoxifen, a selective estrogen receptor modulator (SERM), has been widely used as a conventional medicine for ER positive breast cancer patients [5]. The most prominent obstacle in antiestrogen therapy is the occurrence of drug resistance, and almost ER positive breast cancer patients with advanced disease will develop resistance to endocrine therapy [7]. The death-inducing effects of curcumin in non-endocrine resistant breast cancer cells through multiple pathways, have been well documented [21]. However, in this study, we show that curcumin can also inhibit the growth in tamoxifen-resistant MCF-7/LCC2 and MCF-7/LCC9 cells through multiple mechanisms. Breast cancer cells can induce tamoxifen resistance through upregulation of growth-promoting genes (cyclin D1 and c-Myc) and inhibition of Bcl-2 expression [12,14]. Indeed, we found higher levels of cyclin D1 and c-Myc and lower levels of Bcl-2 in tamoxifen-resistant MCF-7/LCC2 and MCF-7/LCC9 cells compared with the non-resistant cells. Since curcumin inhibited the proliferation in these resistant cells, it is apparent that the changes of these pathways do not affect the activity of curcumin. On the contrary, curcumin suppressed the gene expression of cyclin D1,c-Myc and Bcl-2 in both protein and mRNA levels in the resistant cells. These data suggest that curcumin has the potential to kill the antihormonal resistance breast cancer cells.

NF-κB pathway has been reported, during the last years in several studies, to play an important role in the antiestrogen resistance phenotype [8,22]. In our study, we examined the molecular changes in NF-κB pathway and compared the effect of curcumin on NF-κB pathway members in endocrine- resistant and sensitive breast cancer cells. In our model, we found unchanged expression of p65 compared with MCF-7 cells, not in line with previous results showing increased expression of p65 in tamoxifen/fulvestrant resistant cells [23]. Furthermore, we investigated the phosphorylation level of p65. We demonstrated increased level of p65 phosphorylation at Ser536 in MCF-7/LCC9 but not in MCF-7/LCC2 cells compared with MCF-7 cells, which has been shown to enhance the transactivation potential of p65 [24]. Our data implies MCF-7/LCC2 and MCF-7/LCC9 resistant cells display different modes of dependence on the NF-κB pathway and curcumin could inhibit the total p65 expression in MCF-7 and MCF-7/LCC2 cells, but not significantly in MCF-7/LCC9 cells. However, curcumin suppressed activation of p65 at Ser536 remarkably in MCF-7/LCC9 cells. Because IKKs and IκBα is required for NF-κB activation, we also examined the IKKs and IκBα with treatment of curcumin. Decreased expression of IKKα and IKKβ and IκBα degradation could be observed, combined with the downregulation of total p65, was not consistent with other researches suggesting that curcumin only suppressed the phosphorylation of IKKs and NF-κB p65 in breast cancer cells [20,25,26]. However, the total effect of the dual inhibition of NF-κB pathway may contribute to the inhibition of proliferation in endocrine resistant cells, endocrine-sensitizaiton in wild-type cells and resistance reversal in endocrine resistant cells to tamoxifen. In our model, dynamic changes in IκBα expression appeared under the different curcumin concentrations. At the concentration of 10 μM, curcumin inhibited the expression of IKKs, leading to the reduction of degradation of IκBα. However, when the concentration of curcumin reached to 30 μM, inactivation of p65 may cause downregulation of IκBα that is an NF-κB-regulated gene. Previously, others have reported the preclinical models of acquired endocrine resistance display elevated levels of activity of Src/FAK complex and pharmacological inhibition of Src in endocrine resistant cells appears to enhance the response to tamoxifen [10,27,28]. As previous studies have shown, our study also found upregulation of FAK and higher levels of phosphorylation of Src, which could be suppressed significantly by treatment of curcumin in a concentration dependent manner in MCF-7/LCC2 and MCF-7/LCC9 cells, in consistent with studies suggesting the inhibition of curcumin on Src/FAK in other cells [29,30,31]. Such data reflected curcumin may target the Src pathway to affect biological actions of endocrine resistant cells.

Akt/mTOR, playing a central role in regulation of multiple critical cellular function including cell growth and survival, has been associated with a negative phenotype and high levels of activated Akt/mTOR have been associated with hormonal resistance in breast cancer [11,32]. Indeed some studies have evaluated the effectiveness of Akt/mTOR targeted therapy [33] and found inhibition of Akt/mTOR may restore sensitivity to hormonal based therapies [34]. In the studies present in this report, significantly increased expression of activated Akt could not been found in MCF-7/LCC2 and MCF-7/LCC9 compared with parental cells. In contrast, resistant cells showed increased mTOR phosphorylation on Ser 2488. Recently, it has been reported that curcumin suppresses Akt/mTOR signaling in various cancers including breast cancer [35,36,37]. However, in resistant cells, the effects of curcumin on the signaling of Akt/mTOR have not been explored. In the present study, we firstly demonstrated that curcumin inhibited the phosphorylation of Akt /mTOR as well as downstream targets p70S6K, S6 and 4EBP1 in a similar concentration-dependent manner in resistant breast cancer cells as in sensitive cells. We believe the extensive effects of curcumin on sensitive and non-sensitive cells is partially due to the inhibition of Akt/mTOR pathway.

EZH2, the key component of Polycomb repressor complex 2 (PRC2), is a histone methyltransferase for H3K27 trimethylation (H3K27me3) [38]. EZH2 has been suggested to be linked to highly proliferative and aggressive behavior and negative prognosis of breast cancer [39,40] and tamoxifen resistance acquisition in ER positive breast cancer [41,42]. Our data showed elevated levels of EZH2 expression in MCF-7/LCC2 compared with that in MCF-7 cells, but no change in MCF-7/LCC9 cells. Furthermore, our results demonstrated that curcumin could induce the downregulation of EZH2 in MCF-7/LCC2 and MCF-7 cells in a dose-dependent manner, but we did not observe the similar effect in MCF-7/LCC9 cells after curcumin treatment. Hua *et al*. found inverse trends between EZH2 expression and ERK1/2 phosphorylation and curcumin inhibited EZH2 expression by activation of MAPK pathway including ERK1/2, JNK and p38 in MDA-MB-435 [43]. However, in MCF-7/LCC9 in our model, both EZH2 and phospho-ERK1/2 were undetectable, revealing unique modulating mechanisms of EZH2 different from that in MCF-7/LCC2. More importantly, in different cell context, curcumin had varied regulation of JNK and ERK [44,45], which may also lead to kinds of change models of EZH2. Our results indicated that curcumin may epigenetically modulate the proliferation and endocrine resistance in ER positive breast cancer cells. 

## 3. Experimental

### 3.1. Cell Culture and Chemicals

The human breast cancer cell lines MCF-7, MCF-7/LCC2 and LCC 9 were kindly provided by Dr Robert Clarke (Georgetown University Medical Center, Washington, DC, USA). MCF-7/LCC2 and MCF-7/LCC9 cells are tamoxifen and tamoxifen/fulvestrant-resistant cell lines [46,47]. All cells were routinely maintained in MEM containing phenol red and supplemented with 5% fetal bovine serum (FBS) (Invitrogen, Carlsbad, CA, USA), in a humidified 37 °C incubator containing 5% CO_2_. 4-hydroxytamoxifen (4-OHT) and curcumin were purchased from Sigma-Aldrich (St. Louis, MO, USA), dissolved in DMSO at a stock concentration of 10 mM and 100 mM.

### 3.2. Sulforhodamine B (SRB) Assay

Cells were seeded in each well of the 96 well plates. After culture, 200 μL cooled trichloroacetic acid per well was added and incubated for 60 min in at 4 °C. The plates were then washed with distilled water and dried. SRB solution (150 μL) at 0.4% (w/v) in 1% acetic acid was added and incubated for 30 min at RT. The well plates were then washed with 1% acetic acid and dried. 100 μL 10 mM Tris base was added to the wells to solubilize the bound SRB, and absorbance was then read at 560 nm on an automated microplate reader (VERSAmax, Molecular Devices, Sunnyvale, CA, USA). The relative survival (%) was calculated using the following equation: (A560/A560 control) × 100%. The experiments were performed at least three times, with each condition plated in triplicate.

### 3.3. Drug Treatment Assay

For curcumin treatment, cells were plated into 96-well plates at 3000/well density and grown in regular media for 24 h. Cells were then incubated with varying concentrations of curcumin for 24 h, 48 h, 72 h and 96 h. For the tamoxifen response assay, 2,500 cells per well were plated in 96-well plates in phenol red-free IMEM supplemented with 5% charcoal dextran-treated FBS (Tissue Culture Biologicals, Los Alamitos, CA, USA) for 24 h and then incubated with combination of 2.5 μM curcumin and varying concentrations of OHT for 3 days. Media/tamoxifen mixes were given for the next 4 days. Survival fraction was measured using SRB.

### 3.4. Colony Formation Assay

Cells were plated 500 per well in complete media in six-well plates (Corning, Acton, MA, USA) and allowed to adhere for 24 h. The next day cells were treated with curcumin (10 and 30 μM) and equal volumes of DMSO. After 24 h, curcumin-containing media were removed, and cells were allowed to form colonies in complete media for 14 days. And then, the colonies were fixed with a solution of acetic acid and methanol (1:3) for 15 min, stained with 0.5% crystal violet for 30 min and counted manually.

### 3.5. Cell Cycle Analysis

All cells, after treated with 30 μM curcumin for 12 h, were harvested and washed twice with cooled PBS and fixed in 75% ethanol for 2 h at 4 °C. The fixed cells were washed with cooled PBS and were then stained with the staining solution containing 0.05 μg/mL PI (Sigma-Aldrich), 1 μg/mL DNase-free RNase (Sigma-Aldrich) for 30 min at RT. Ten thousand events were acquired using a FACSCalibur analyzer (Becton-Dickinson, San Jose, CA, USA), and cell cycle data were determined using Modfit software (Verity Software House, Topsham, ME, USA).

### 3.6. Apoptosis Analysis

After treatment of curcumin at concentration of 10 μM and 30 μM, cells were detached with EDTA-free trypsin and washed twice with cooled PBS. Cells were resuspended in 400 μL 1× loading buffer with 5 μL Annexin V and 5 μL PI (BD Pharmingen, San Diego, CA, USA) for 15 min on ice in dark. Analyses were performed at FACSCalibur analyzer (Becton-Dickinson).

### 3.7. Reverse Transcription-Polymerase Chain Reaction Assay

Total RNA was extracted from cells using TRIzol (Invitrogen). One microgram of total RNA was reverse-transcribed into cDNA using Prime Script RT master Mix (Takara, Dalian, China). The cDNA was then amplified and quantified by SYBR Green PCR kit (Takara) with ABI 7500fast system (Applied Biosystems, Foster City, CA, USA). Primers used in this study were as follows: GAPDH: 5'-ACCCAGAAGACTGTGGATGG-3' and 5'-TCTAGACGGCAGGTCAGGTC-3';cyclin D1: 5'-GCTGCGAAGTGGAAACCATC -3' and 5'-CCTCCTTCTGCACACATTTGAA-3';c-Myc: 5'-GGCTCCTGGCAAAAGGTCA-3' and 5'-CTGCGTAGTTGTGCTGATGT-3'; Bcl-2: 5'-GTCTGGGAATCGATCTGGAA-3' and 5'-AATGCATAAGGCAACGATCC-3'; Bcl-xLl:5'-TCTGGTCCCTTGCAGCTAGT-3' and 5'-TCCTTTCTGGGGAAGAGGTT-3'.

### 3.8. Western Blot Analysis and Antibodies

Cells were harvested and lysed in RIPA buffer (Beyotime Institute of Biotechnology, Beijing, China) and protease inhibitor (Roche Applied Science, Indianapolis, IN, USA). Protein concentration was determined using the BCA Protein assay (Beyotime). Protein sample were separated using SDS-PAGE and transferred to PVDF membranes (Millipore, Bedford, MA, USA). Membranes were then incubated with respective primary antibodies, followed by HRP-conjugated secondary antibodies. Proteins were visualized using the chemiluminescence system (GE Healthcare, Piscataway, NJ, USA). The primary antibodies used were cyclin D1, c-Myc, p21, p65, p-p65 (Ser536),4EBP1,p-4EBP1 (Thr37/46), S6, p-S6 (Ser235/236), p70S6k, p-p70S6K (Thr389), Akt, p-Akt (Thr308), Erk1/2, p-Erk1/2 (Thr202/Tyr204) and EZH2 (Cell Signaling Technology, Danvers, MA, USA), Bcl-2, Bcl-xL and FAK (Epitomics, Burlingame, CA, USA), IKKα, IKKβ and IκBα (Santa Cruz Biotechnology, Santa Cruz, CA, USA), Src, p-Src(Tyr418), mTOR and p-mTOR (Ser2488) (Abcam, Cambridge, MA, USA). 

### 3.9. Statistics

All quantified data from each assay were represented as mean ± SD or as indicated. Statistical significant was analyzed by the student’s *t* test, and *p*-values < 0.05 was considered significant for all tests. Statistical analysis was performed using GraphPad Prism Software 5.0.

## 4. Conclusions

Collectively, these studies provide mechanistic evidence for proliferation inhibition of curcumin in endocrine resistant breast cancer cells and suggest that use of curcumin as an alternative combination therapy to enhance tamoxifen actions in wild-type cells and to overcome endocrine resistance in endocrine-resistant cells:MCF-7/LCC2 and MCF-7/LCC9. Our findings demonstrate that curcumin mediates these effects through (i) downregulation of pro-growth and antiapototic molecules, (ii) inhibition of NF-κB and Akt/mTOR signaling pathways, (iii) suppression of Src/FAK complex, (iv) downregulation of EZH2 proteins. These data further provide a persuasive rationale to design clinical trials of curcumin alone and in combination with tamoxifen in breast cancer patients with a poor response to endocrine therapy. However, further experiments in animal models are needed to thoroughly clarify the potential of curcumin in endocrine-resistant breast cancer.

## Supplementary Files

Supplementary File 1 Supplementary Material.pdf (PDF, 137 KB)
