# Supplementary material for: Curcumin Induces Cell Death and Restores Tamoxifen Sensitivity in the Antiestrogen-Resistant Breast Cancer Cell Lines MCF-7/LCC2 and MCF-7/LCC9
Source: Molecules. 2013 Jan 8;18(1):701–20. doi: 10.3390/molecules18010701 (PMC6269686; doi:10.3390/molecules18010701)

Supplementary Information

Figure S1. Curcumin exerts minor effects on HBL-100. (**A**) HBL-100 cells were treated with varying concentrations of curcumin, cell proliferation and IC50 were determined by SRB assay on days 1, 2, and 3. Each value represents the mean ± SD (n = 3); (**B**) HBL-100 cells were incubated with curcumin (10 and 30 μM) for 24 h, followed by staining with Annexin-V/PI; (**C**) HBL-100 cells were treated with 30 μM for 12 h and DNA content was analyzed by flow cytometry; (**D**) HBL-100 cells were treated with combination of increasing concentrations of OHT and 2.5 μM curcumin for 3 days and OHT alone for the next 4 days. Drug response was determined by SRB assay. Each value represents the mean ± SD (n = 3).

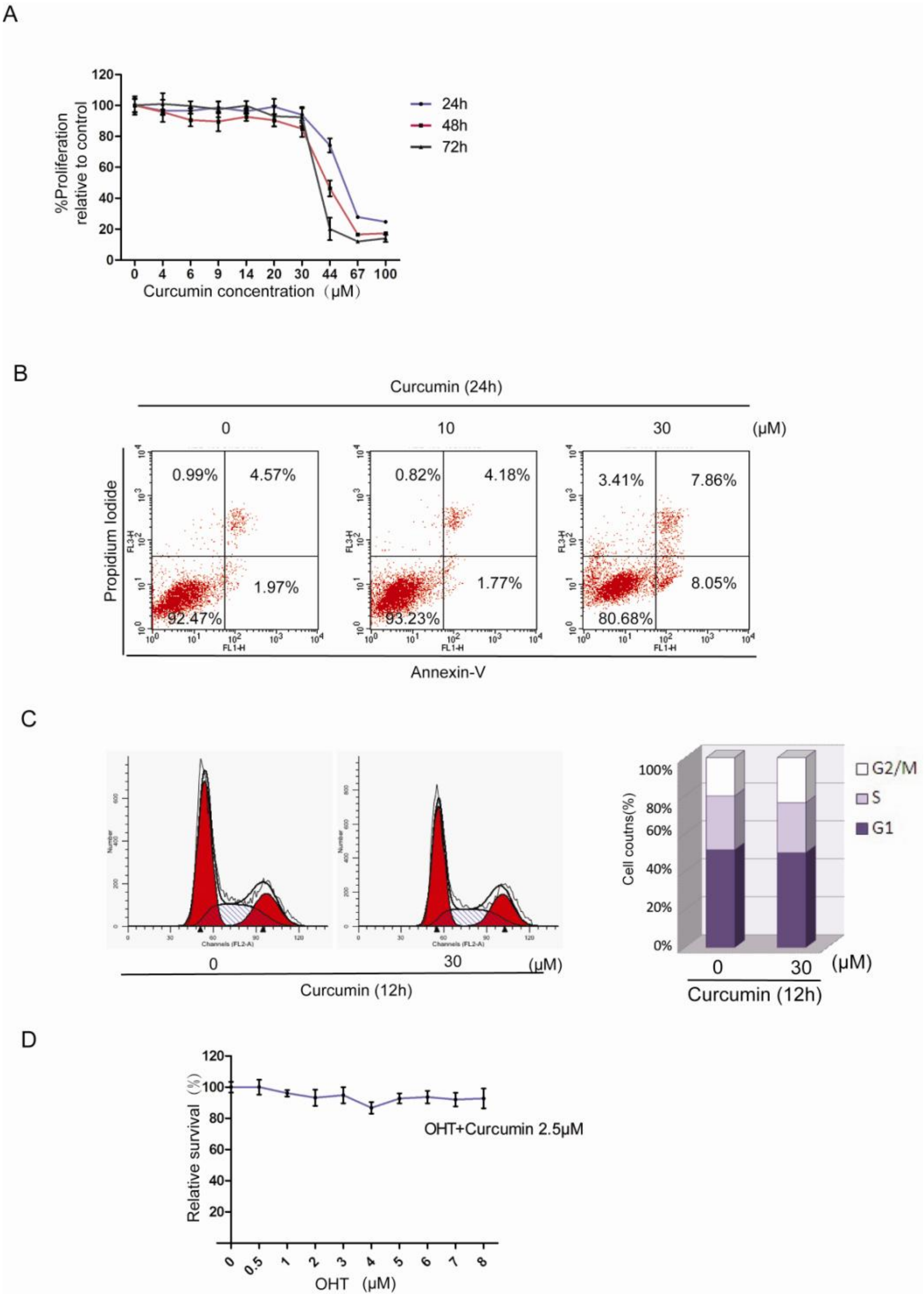

Supplement: Supplementary File 1 — Supplementary Material.pdf (PDF, 137 KB) [file molecules-18-00701-s001.pdf]
